# A Novel Method for Inducing Nerve Growth via Modulation of Host Resting Potential: Gap Junction-Mediated and Serotonergic Signaling Mechanisms

**DOI:** 10.1007/s13311-014-0317-7

**Published:** 2014-12-02

**Authors:** Douglas J. Blackiston, George M. Anderson, Nikita Rahman, Clara Bieck, Michael Levin

**Affiliations:** 1 Center for Regenerative and Developmental Biology and Department of Biology, Tufts University, 200 Boston Avenue, Suite 4600, Medford, MA 02155 USA; 2 Yale Child Study Center and Department of Laboratory Medicine, Yale University School of Medicine, 230 S. Frontage Rd., New Haven, CT 06519 USA

**Keywords:** Membrane potential, serotonin, innervation, *Xenopus*, ion channel

## Abstract

A major goal of regenerative medicine is to restore the function of damaged or missing organs through the implantation of bioengineered or donor-derived components. It is necessary to understand the signals and cues necessary for implanted structures to innervate the host, as organs devoid of neural connections provide little benefit to the patient. While developmental studies have identified neuronal pathfinding molecules required for proper patterning during embryogenesis, strategies to initiate innervation in structures transplanted at later times or alternate locations remain limited. Recent work has identified membrane resting potential of nerves as a key regulator of growth cone extension or arrest. Here, we identify a novel role of bioelectricity in the generation of axon guidance cues, showing that neurons read the electric topography of surrounding cells, and demonstrate these cues can be leveraged to initiate sensory organ transplant innervation. Grafts of fluorescently labeled embryological eye primordia were used to produce ectopic eyes in *Xenopus laevis* tadpoles. Depolarization of host tissues through anion channel activation or other means led to a striking hyperinnervation of the body by these ectopic eyes. A screen of possible transduction mechanisms identified serotonergic signaling to be essential for hyperinnervation to occur, and our molecular data suggest a possible model of bioelectrical control of the distribution of neurotransmitters that guides nerve growth. Together, these results identify the molecular components of bioelectrical signaling among cells that regulates axon guidance, and suggest novel biomedical and bioengineering strategies for triggering neuronal outgrowth using ion channel drugs already approved for human use.

**Electronic supplementary material:**

The online version of this article (doi:10.1007/s13311-014-0317-7) contains supplementary material, which is available to authorized users.

## Introduction

The field of regenerative medicine has made remarkable progress in the wake of the molecular revolution, owing largely to cross-disciplinary approaches and growing convergence between developmental biology, neurobiology, bioengineering, and genetics. Already visible on the horizon are a number of exciting new treatments for a variety of human nervous system impairments, including paralysis, deafness, and blindness. Clinical trials are underway for a range of sensory augmentation devices, including retinal prosthetics and a new generation of cochlear implants [1–6], and recent findings include the generation of full vertebrate eyes in culture or *in vivo* [7–9].

The regeneration of damaged sensory structures such as eyes, or the implantation of bioengineered sensory (or even effector) components, requires afferent innervation of the host for information to be transmitted. In natural development, these connections are orchestrated by a suite of overlapping attractive and repulsive secreted signals that direct axon pathfinding to particular targets. In the case of the eye these signals include Sonic Hedgehog, Ephrin, Netrin, Semaphorin, and Slit family members, with various combinations being required for the exit of retinal ganglion cells from the base of the optic cup, the journey of the neurons from the eye to the brain, the crossing of the 2 optic nerves at the optic chiasma, and, finally, penetration of the visual center within the brain [10–14]. While the pathways involved in retinal ganglion pathfinding have been well documented in the context of early development, how eyes respond to these signals when they are spatially or temporally perturbed is not understood. Answering this question is essential for regenerative medicine approaches to augment, exchange, or replace sensory systems in an already developed organism, or to understand and repair birth defects affecting the visual system.

Insight may be gained from a number of studies in which researchers create morphologically complete ectopic eyes in vertebrates through the transplantation of developing eye primordia [15–19], misexpression of a master eye control genes, including *Otx2*, *Pax6*, *Rx1*, and *Six3* [20–25], or establishment of eye-specific bioelectrical states in embryonic tissues [9]. Cellular tracking revealed that in some cases the retinal ganglion cells of ectopic eyes penetrated the host, growing toward the brain or spinal cord [16, 26], though the signals guiding the transplanted neurons have yet to be identified. Further, in at least one species, *Xenopus laevis*, it has been shown that animals can use these ectopic eyes in color learning assays, even when they are located far from the head of the individual [27–29]. The mechanism underlying this observation currently remains unclear, however, and a major goal is to determine what signals instruct ectopic eyes to innervate specific targets so they could be leveraged in molecular and regenerative medicine.

Bioelectricity is known to be a powerful instructive signal that cells and tissues use in a surprisingly diverse array of developmental and regenerative contexts [30–33], and large literature exists on the ability of extracellular electric fields, both endogenous and exogenous, to direct neuronal growth and orientation [34–48]. The ability of another aspect of cell biophysics—resting potential gradients—to dictate tissue identity and large-scale organization has begun to be exploited but has not yet been applied to the control of innervation *in vivo* [49, 50]. While the mechanistic studies of electric field effects on neurons have focused on the neuronal cells themselves, there have been no investigations of how *V*_mem_ of the neurons *environment* may affect their outgrowth decisions.

Here, we asked whether and how the bioelectric topography of the host body could be leveraged to regulate the innervation emerging from ectopically transplanted organs. Our goals were to: 1) test the specific hypothesis that innervation from ectopic implants was sensitive to the resting potential states of cells in the microenvironment; 2) determine the molecular mechanisms by which this signaling occurs; and 3) establish a proof-of-principle of using ion channel drugs to modulate nerve growth after microsurgery. Using eye primordium grafts in *Xenopus* embryos, a model system that greatly facilitates insight into mechanisms of neurodevelopmental pathways [51], ectopic eyes were produced at caudal locations. Lineage tracers marking the donor tissue revealed that when host tissues’ *V*_mem_ was depolarized, these ectopic eyes drastically hyperinnervated the host, extending axons through the majority of the fin and trunk of the developing tadpole. Functional experiments probing the downstream mechanism underlying the phenotype revealed serotonin (5-hydroxytryptamine, 5-HT) signaling (a charged neurotransmitter molecule known to be transported between cells along voltage gradients) to play an essential role in mediating the hyperinnervation effect. These results have a number of implications for both sensory and regenerative biology, revealing the mechanistic details of a novel growth control pathway for ectopic nerves that may underlie behavioral brain–body plasticity, and identifying a new endogenous role for embryonic 5-HT. The ability to control axon outgrowth and attraction through manipulation of membrane voltage suggests new approaches for guiding neurons across damaged areas, directing neurons to specific targets within the host, and promoting afferent innervation by bioengineered sensory implants. Such approaches have the potential to increase the efficacy of treatments for a wide range of nervous system impairments, and to extend the applicability of existing electroceuticals [52, 53].

## Materials and Methods

### Animal Husbandry

*Xenopus laevis* embryos were obtained through *in vitro* fertilization according to standard protocols [54]. Animals were reared in 0.1× Marcs Modified Ringer solution (MMR), pH 7.8, at 22 °C for the first 24 h (prior to surgery) and 16 °C thereafter. All animals were raised in a 12 h : 12 h light : dark cycle and were staged according to Nieuwkoop and Faber [55]. Animal density was a maximum of 30 individuals per 100 ×25 mm Petri dish, with media exchanges occurring every 3 days. For experiments involving high chloride media, 0.1× MMR was supplemented with additional NaCl to raise the chloride level from the 10.5 mM base level to a final concentration of 65 mM. This treatment has been previously been shown to raise the intracellular concentration of chloride following ivermectin exposure [56]. All experimental procedures involving *Xenopus* embryos were approved by the institutional animal care and use committees and Tufts University Department of Laboratory Animal Medicine under protocol M2011-70

### Microinjection

Fluorescent donor animals were created through microinjection of capped synthetic tdTomato mRNA [57]. mRNA was synthesized using standard message machine kits (Life Technologies, Carlsbad, CA, USA) and injected into animals in 3 % Ficoll solution using a pulled capillary, targeting all 4 cells of a stage 3 embryo to maximize expression. Three hours after microinjection the embryos were washed in 0.1× MMR and returned to the incubator for 24 h, at which time they were sorted for expression of the fluorescent construct using an Olympus BX-61 microscope (Olympus, Tokyo, Japan) equipped with a Hamamatsu ORCA AG CCD camera (Hamamatsu, Shizuoka-ken, Japan). The hyperpolarizing channel Kir4.1 has been described previously [58], as has the dominant negative gap junction construct 3243H7 (H7) [59, 60].

### Microsurgery

Prior to microsurgery, the vitelline membranes of stage 23 tdTomato^+^ donors and wild-type (WT) recipients were removed and the embryos anesthetized in a 0.02 % tricaine solution, pH 7.5, in 0.1× MMR. Using surgical forceps, the presumptive eye primordium was excised from tdTomato+ donors with care taken to avoid any underlying or surrounding tissue. Using the same forceps, a small slit was created in the posterior tissue of the WT recipient, directly ventral to the neural tube. The donor tissue was then gently inserted into this wound and allowed to heal for 10 min. To avoid any confounding effects of laterality on transplants, only the left eye was harvested from the donor, and all recipients received grafts to their left sides. In addition, the transplant was positioned in its natural proximal/distal orientation, that is with the eye facing “outwards” from the body of the animal. Transplanted tissue generally fused with the host within minutes and animals were washed 2× in fresh 0.1× MMR before being returned to an incubator at 16 °C. To generate eyeless tadpoles, stage 34 animals were anesthetized for 10 min in 0.02 % tricaine solution. Surgical forceps were used to create a small opening in the epidermis overlying the eye, after which the entire structure was removed. Operated animals were kept anesthetized for 30 min before being washed twice with 0.1× MMR and returned to an incubator at 16 °C.

### Immunohistochemistry

Visualization of host innervation was performed by whole mount immunohistochemistry [61]. Animals were fixed overnight in MEMFA [100 mM 3-(N-morpholino)propanesulfonic acid (pH 7.4), 2 mM ethylene glycol tetraacetic acid, 1 mM MgSO_4_, 3.7 % (v/v) formaldehyde], washed in phosphate buffered saline Tween-20 (PBST), and blocked for 1 h at room temperature with 10 % normal goat serum in PBST. Samples were then rocked overnight at 4 °C in monoclonal antiacetylated alpha-tubulin antibody (Sigma T7451; Sigma, St. Louis, MO, USA), diluted 1 : 500 in PBST +10 % goat serum. Following primary exposure, samples were washed 3 times for 15 min in PBST before a 60-min secondary incubation with AlexaFlour-555 conjugated secondary at 1 : 1000 diluted in PBST. Following secondary incubation, samples were washed 3 times for 15 min in PBST and imaged on an Olympus BX-61 microscope.

### Pharmaceutical Exposure

Animals were exposed to pharmaceutical compounds immediately following surgery, stage 24, through imaging at stage 46. The only exception was *para*-chlorophenylalanine, in which case the embryos were exposed directly after fertilization, throughout their entire development, and during surgical procedures. All pharmaceuticals were diluted from stock solutions in dimethyl sulfoxide and were refreshed every 3 days. For the indicated stages, animals were exposed in 0.1× MMR to altanserin, 10 μM; cyanopindolol, 50 μM; fluoxetine, 10 μM; ivermectin, 1 μM; lindane, 280 μM; metergoline, 3.3 μM; *para*-chlorophenylalanine, 12.5 mM; SB258719, 50 μM; 5-HT, 5 mM; tricaine mesylate, 318 μM; and tropisetron, 10 μM. Drug concentrations used in the present study were determined through toxicity screens and were applied at levels that did not result in lethality or observable developmental defects.

### Imaging

To image transplanted eye innervation of the host, animals were first anesthetized for 10 min in 0.02 % tricaine solution, pH 7.5, after which they were transferred individually to depression slides and photographed on an Olympus BX-61 microscope. Donor fluorescence was detected with a tetramethylrhodamine filter set (Olympus U-MRFPHQ), which was then overlaid with standard bright field images. Animals were imaged at both 40× and 100× magnifications, and raw widefield stacks were used to resolve the regions of interest.

### Tissue 5-HT Analysis

Tissue concentrations of 5-HT were determined by high-performance liquid chromatography with fluorometric detection using a modification of a published methods for the determination of 5-HT in blood and brain [62, 63]. Briefly, animals (*n* =1–10 per tube) were sonicated (two or three 1-s pulses, medium power) in 100–500 μl of an ice-cold solution containing 1 % ascorbic acid and 10 ng/ml of the internal standard N-methylserotonin. Following perchloric acid deproteinization (10 % volume of 3.4 M HClO_4_), samples were centrifuged at 10,000 × *g* and the supernatants directly injected on the high-performance liquid chromatography system.

### Statistics

Statistical analyses were performed using Prism v. 5 (GraphPad Software, La Jolla, CA, USA). All data were collected as binomial outputs (presence or absence of a phenotype) and analyzed using nonparametric statistics. For comparisons between control and treated animals, Fisher’s exact tests were performed. In the cases where multiple comparisons were made within an experiment (including the analysis of 5-HT and gap junction blocker results) Bonferroni corrections were employed to correct for experiment-wide α level. To quantify the innervation in control and ivermectin-exposed animals, Sholl analysis was performed on raw widefield image stacks from each treatment using ImageJ v. 1.49 (NIH, Bethesda, MD, USA). After processing with a threshold algorithm, concentric circles with and increasing radius of 50 μm were drawn starting at the region of image closest to the ectopic eye and the axons crossing each radius counted using an automated function. Analysis began 200 μm from the center of the circle to reduce noise due to over exposure of fluorescence near the ectopic eye itself. Comparisons between control and treated animals were performed by two-way analysis of variance.

## Results

### Chloride Channel Activation Induces Hyperinnervation from Ectopic Eyes

**Fig. 1:**
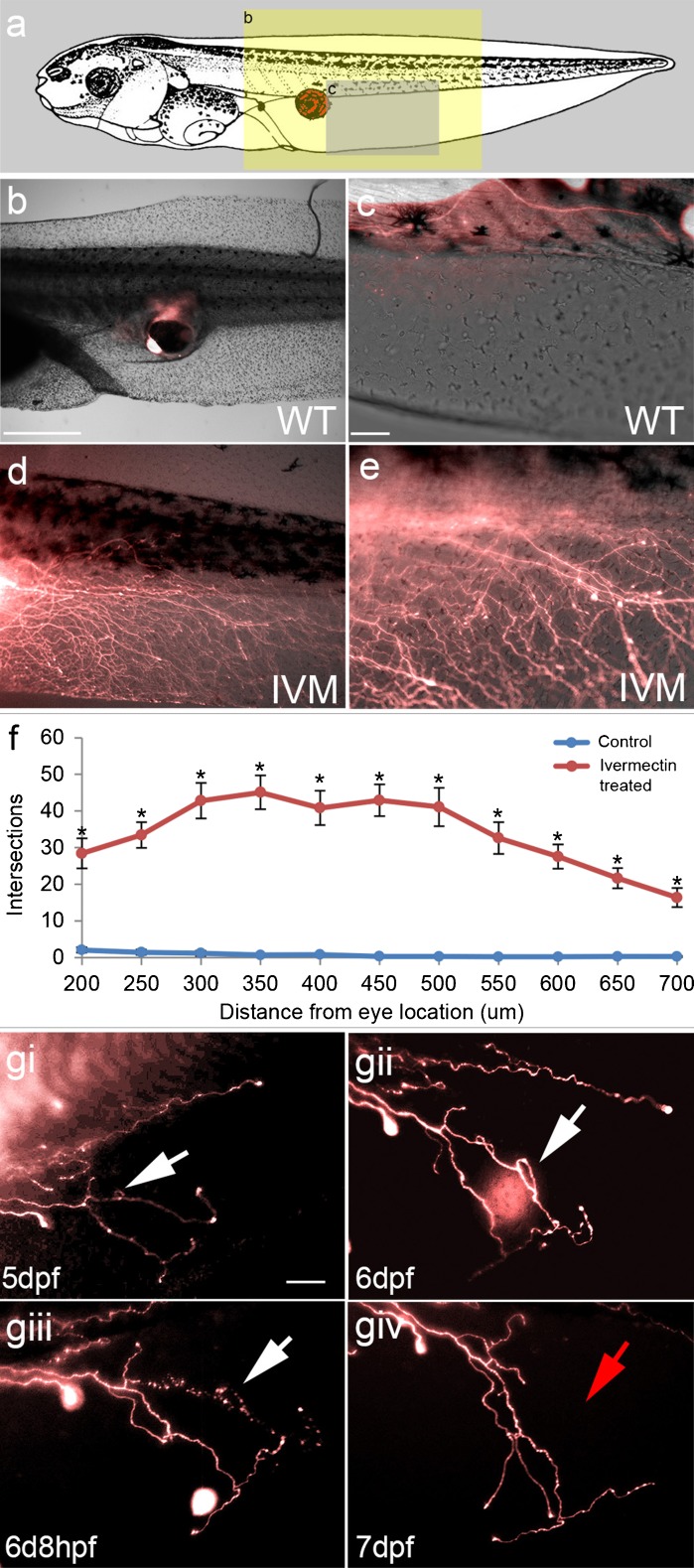
Ectopic eyes hyperinnervate hosts in response to chloride channel activation. (a) Diagram of eye primordium graft location (red eye) and location imaged for innervation analysis (yellow shaded area =40×; gray shaded area =100×). (b) Eye primordium removed from a donor tdTomato-expressing *Xenopus* and transplanted to caudal locations of a wild-type (WT) recipient produces ectopic eyes at the site of the graft (scale bar =500 μm). (c) Fluorescent labeling of donor tissue reveals that ectopic eyes occasionally innervate the host, with small numbers of axons observed in the fin and trunk of the tadpole (scale bar =100 μm). (d), In the presence of the glutamate-gated chloride channel activator ivermectin (IVM), ectopic eyes hyperinnervate the host, with labeled axons present throughout large portions of the fin and trunk of the recipient. (e) High magnification reveals extensive branching of donor axons in response to IVM. (f) Sholl analysis comparing WT (*n* =15) and IVM-exposed (*n* =14) embryos reveals significantly more axons (*P* <0.01) in treated animals at both proximal and distal locations to the transplanted eye. (g) Time lapse imaging of ectopic eye innervation shows extensive axon remodeling in response to IVM, including extension, retraction, and degradation over 4 days. Loopback structures were observed in which axons appear to self-cross, after which the axon shows blebbing and degradation (white arrows; scale bar =100 μm). Error bars indicate ±1 SEM. Asterisks represent values which significantly differ from controls (2-way analysis of variance, followed by Bonferroni post hoc analysis)

To begin to investigate the bioelectrical cues that guide innervation of ectopic organs, we established an assay in *X. laevis*: eye primordium transplants [27], which result in complete eyes developing at the site of the graft. Eyes could be transplanted rostrally or caudally, with success in >95 % of performed microsurgeries, and were shown through behavioral analysis to be functional [27, 29]. To visualize any innervation arising from donor tissue in the host, eye primordium was taken from animals ubiquitously expressing the fluorescent protein tdTomato. Fluorescence revealed that ectopic eyes created in the tails of *Xenopus* tadpoles often resulted in no innervation of the host, or in some cases small amounts of innervation in the tail or trunk of the animal (Fig. 1a–c).

To test the functional roles of polarization state of the host tissue in determining the quality of innervation, we took advantage of the chloride channel activator ivermectin (targeting glycine gated chloride channel, glutamate gated chloride channel, and gamma-aminobutyric acid (GABA) A receptor family members), which depolarizes cells expressing these channels through chloride efflux upon binding [56, 64]. Application of ivermectin after surgery had a dramatic effect on ectopic eye innervation, 37 % of treated animals displayed a drastic hyperinnervation phenotype (*n* =59; *P* <0.01) (Fig. 1d,e), with labeled donor axons present throughout the tailfin and trunk of affected tadpoles. The phenotype appeared to be biphasic, tadpoles showed either little/no innervation, or large amounts of axons were observed covering the entire ventral tailfin. Hyperinnervation was quantified using Sholl analysis: affected animals showed a significant increase in axons at both proximal and distal locations relative to untreated transplanted eyes (2-way analysis of variance; *P* <0.01) (Fig. 1f).

Time-lapse imaging revealed that neurons began to emerge from the graft site 2 days after transplantation at stage 38, and continued to grow through the remainder of the experiment. In addition to growth, neurons demonstrated extensive remodeling throughout the study. A number of individual “loopback” patterns were observed, where axons appeared to reverse direction and cross themselves (Fig. 1gi,ii). In these cases, neuronal blebbing was detected within 24 h of loopback, and within 2 days the entire neuron was degraded (Fig. 1giii,iv). In other cases axons were seen to retract before extending in a different direction. As noted in previous studies of glycine receptor chloride channel-expressing cell depolarization in *Xenopus* [56], the overall development of the host animals was completely normal, though late-stage animals were paralyzed owing to the silencing effect of depolarization on the host musculature.

**Fig. 2:**
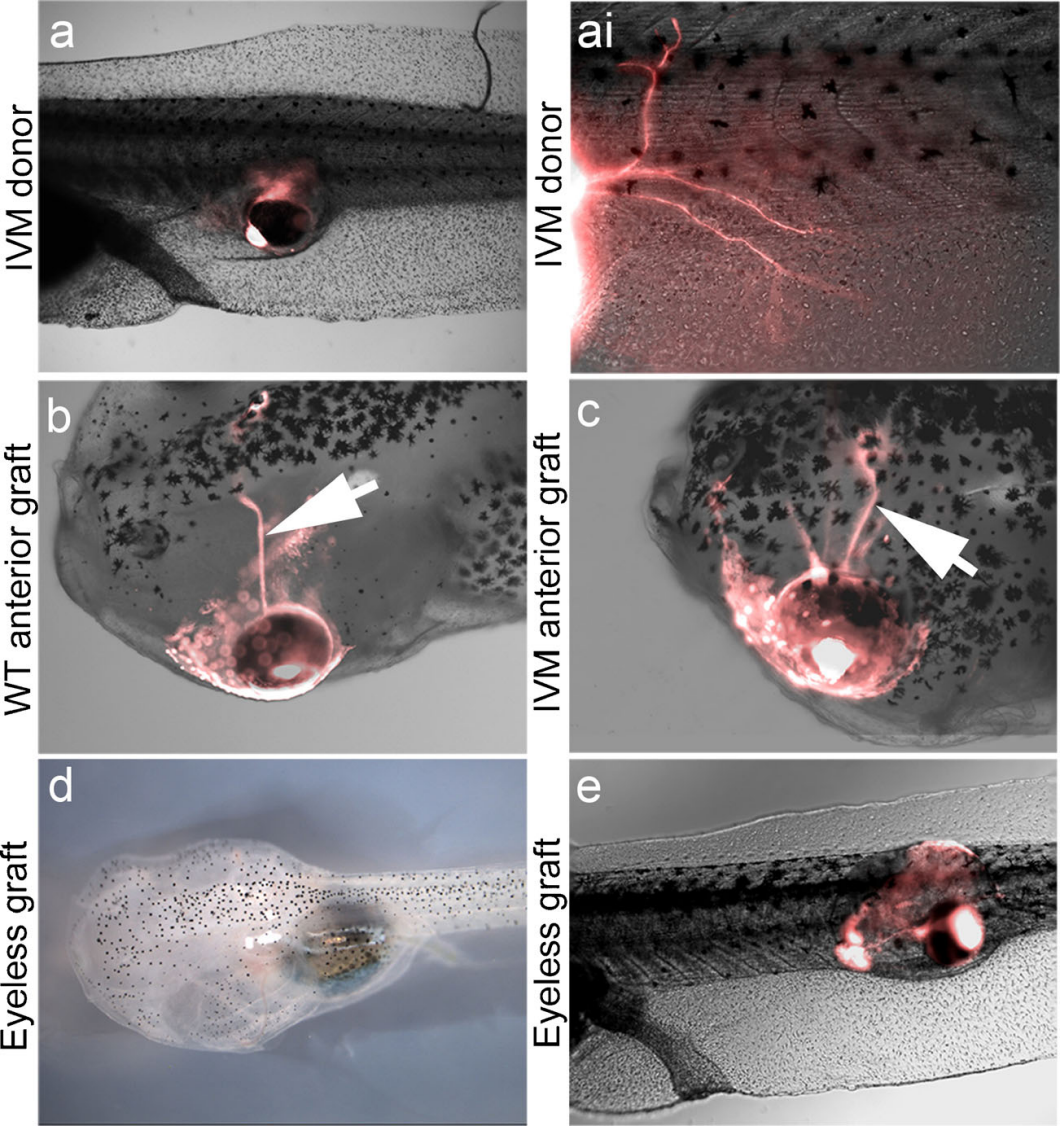
Ectopic eye hyperinnervation is location-specific and requires depolarization of the host. (a) Donor eye primordium treated with ivermectin (IVM) prior to implantation does not result in hyperinnervation of the host (eye is off frame to the left of the image). (ai) High magnification of donor eyes treated with IVM reveals a small number of eyes extend few projections into the host, but not in significant levels on hyperinnervation. (b) When native tadpole eyes are extirpated and replaced with labeled donor eye tissue, optic nerves (white arrow) emerge from replacement structures and target the optic tecta of the host. (c) When native eyes are replaced with labeled donor eyes in the presence of IVM, hyperinnervation is not observed. Transplanted eyes send single optic nerves to the optic tecta of the host (white arrow). (d) *Xenopus* eyes can be surgically removed at tadpole stages, resulting in eyeless animals. (e) Ectopic eyes induced in eyeless animals do not hyperinnervate the host. WT = wild-type

We next asked whether the hyperinnervation was induced by depolarization of the host tissue or the donor tissue. To address this question, fluorescent donors were cultured in medium containing ivermectin from fertilization through the time the primordium was excised, after which the transplant tissue was washed in standard media before being grafted into hosts. Ivermectin is known to be an irreversible activator of chloride channels [65], and ivermectin-exposed tadpoles are paralyzed for days, confirming that treated tissue retains its bioelectrical state long after the drug is removed. Analysis of these transplants revealed that when only donor tissue was exposed to ivermectin, the resultant tadpoles did not display any significant differences in innervation compared with untreated embryos (*n* =32; *P* =0.55 (Fig. 2a). In some cases a small number of axons were observed exiting the ectopic eyes (Fig. 2ai) in both treated and control tadpoles, but animals largely showed no innervation following transplantation, indicating ivermectin exerts its effect not by altering the properties of the ectopic nerves themselves, but by changing the properties of their host environment. Taken together, these data reveal that manipulation of chloride flux can strongly and specifically increase innervation from ectopic organs.

### Chloride Channel Activity-induced Hyperinnervation Distinguishes Native from Ectopic Nerve

Given that ectopic nerve growth was radically altered by ivermectin exposure, we next checked whether the host’s own normal innervation was similarly disrupted. To determine whether the observed phenotype was specific to ectopic nerve, or whether depolarization caused the same degree of hyperinnervation of the native nervous system, immunohistochemistry was performed against the neuronal marker acetylated tubulin. Culturing animals in ivermectin, regardless of whether they possessed ectopic eyes or not, did not result in any detectable large-scale neural patterning defects in the host animal (Supplementary Fig. 1). Remarkably, the native optic nerves, somite patterns, and lateral line all appeared normal and were certainly not affected to the extent observed in transplanted nerves, indicating the effect of ivermectin targets transplanted nerve but not the host’s normal neural anatomy.

Why are ectopic nerves hyperinnervating while the native innervation is unaffected by ivermectin? We first asked if this distinction was due to the surgery (the transplantation history of the ectopic eye tissue) or simply to its aberrant location within the host. To determine if ectopic eye hyperinnervation was location specific, the native eye of a developing tadpole was removed and replaced with labeled donor tissue. In this case, the surgery transplants an eye into its normal native location, and in the absence of ivermectin, donor eyes extend optic nerves to the optic tectum of the host in apparently the same manner as native eyes (Fig. 2b). These optic nerves appeared identical to those of WT animals, and obeyed the same lateralization rules, penetrating the brain hemisphere opposite to the eye location. When the same surgeries were performed in the presence of ivermectin the results were unchanged, transplanted eyes extended optic nerves to the midbrain of the host, and no hyperinnervation was observed (*n* =20; *P* =1.00 (Fig. 2c). These results confirmed that the effect of ivermectin on transplanted eyes was location specific: exposure of eyes in the posterior resulted in hyperinnervation of the host, while those in the anterior, normal eye field, did not.

An additional possibility was that the presence of native eyes suppressed innervation by ectopic structures, and that this suppression was simply alleviated by ivermectin exposure. To test this hypothesis, microsurgeries were performed on stage 30 tadpoles to remove their native eyes. This stage was chosen because time lapse revealed it was prior to the stage when ectopic eyes normally innervate the host. Earlier time periods were ruled out as *Xenopus* tadpoles regenerate eyes lost earlier in development. All microsurgeries performed were successful, producing eyeless tadpoles that did not contain any other detectable developmental defects compared with WT siblings (Fig. 2d). When ectopic eyes were added to eyeless tadpoles at posterior locations, in no cases was hyperinnervation observed (*n* =32; Fig. 2e). In the majority of cases no axons were seen exiting the ectopic eyes, and when labeled neurons were observed they were few in number and length. These results indicated that the presence of native eyes in a tadpole does not inhibit innervation by ectopic eyes.

### Donor Innervation is Regulated by Resting Potential Changes

Exposure to ivermectin depolarizes cells expressing glutamate/glycine-gated chloride channels by locking the targets in an open position, leading to Cl^–^ loss from chloride-rich *Xenopus* cells [56]. To determine if the phenotype caused by ivermectin was a specific effect of chloride signaling (or even an off-target effect of the drug), or was a response to membrane depolarization per se (regardless of which ion’s conductance caused it), experiments were performed to examine ectopic eye innervation in response to voltage changes driven by chloride, potassium, and sodium channels.

**Fig. 3:**
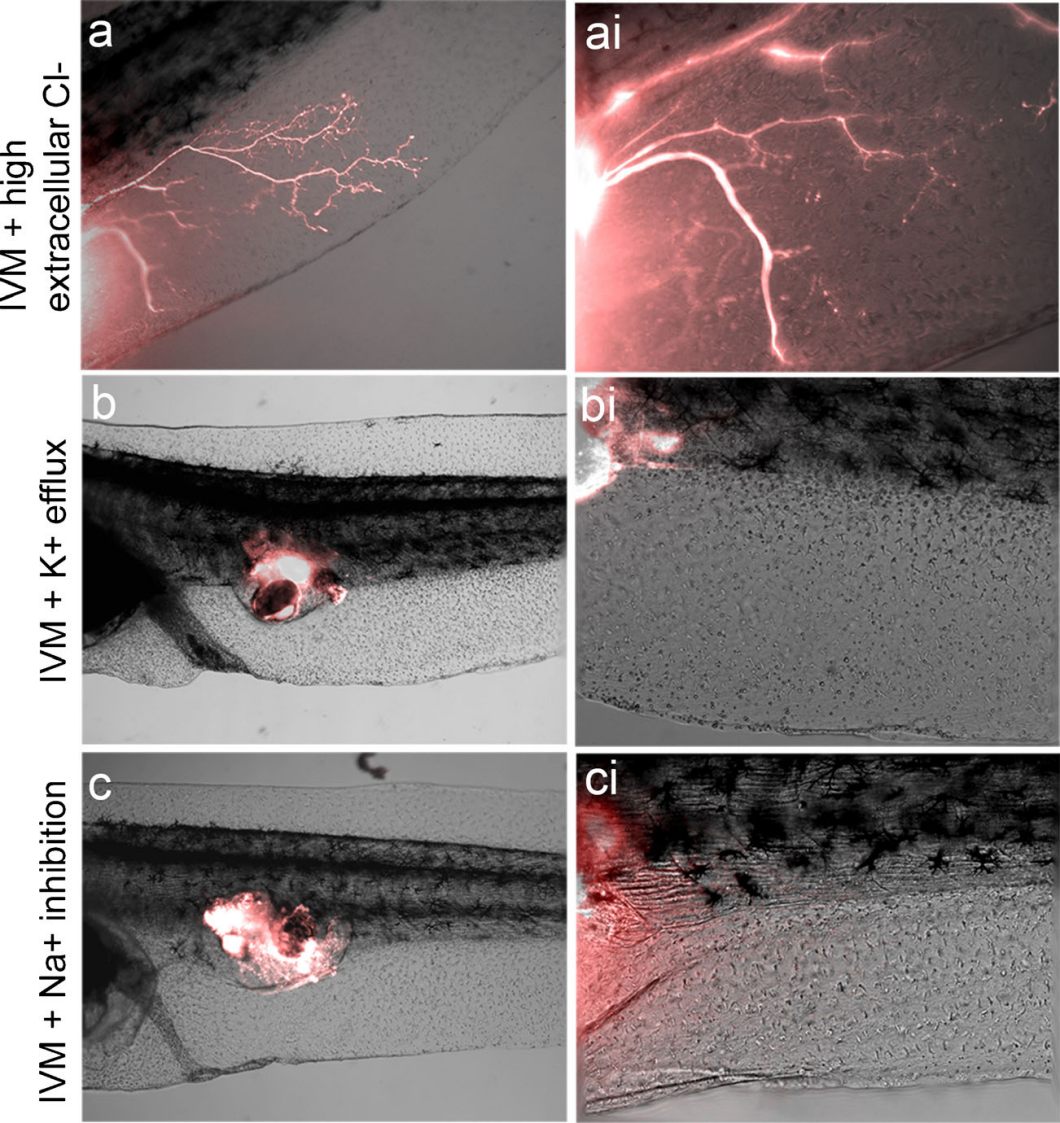
Ectopic eye hyperinnervation is a result of membrane voltage depolarization, not restricted to chloride flux. (a) While ectopic eyes hyperinnervate hosts in response to ivermectin (IVM), supplementing the media with high chloride levels (hyperpolarizing cells affected by the drug) inhibits the extent of innervation and branching pattern. (b) Injecting mRNA coding for the hyperpolarizing potassium channel Kir4.1 inhibits hyperinnervation of ectopic eyes in response to IVM exposure. (c) Treating animals with the sodium transport inhibitor tricaine mesylate, which hyperpolarizes affected cells, inhibits hyperinnervation of ectopic eyes in response to IVM exposure

The intracellular concentration of chloride ions in developing tadpoles can be as high as 60 mM, while that of standard media is 10 mM, leading to a cellular loss of chloride (and membrane depolarization) following ivermectin exposure. To reverse this effect and hyperpolarize GlyCl-expressing cells, a supplemented medium was created with a 65 mM chloride concentration; the same strategy was previously used to characterize the function of the glycine receptor-expressing cells during embryogenesis [56]. Animals were raised in this medium following transplantation (during the same time periods as drug exposure) and host innervation was visualized as in the previous experiments. Imaging revealed that chloride supplemented media significantly inhibited ivermectin induced hyperinnervation of the host [*n* =37 (*P* =0.05); 19 % hyperinnervation in high chloride treatment compared with 37 % with ivermectin only). Most animals showed no innervation, and those in which axons were observed showed a greatly reduced number and simpler branching pattern (Fig. 3a,ai) compared with ivermectin only-treated animals (e.g., Fig. 1d,e). No developmental defects were noted in animals raised in high chloride solutions compared with siblings in standard media, indicating the effect on ectopic eyes was not the result of any large-scale developmental defects. Given the ability of high extracellular chloride to inhibit the hyperinnervating effect of ivermectin precisely as predicted by the Goldman equation, we conclude that the effect is not due to an ion-independent function of ivermectin.

We next asked whether the effect was chloride-specific by attempting to inhibit the ivermectin effect with a hyperpolarizing potassium channel. mRNA encoding the hyperpolarizing Kir4.1 potassium channel [56, 58, 66] was injected into both cells of host embryos at the 2-cell stage for ubiquitous expression. At stage 24, labeled eye primordia were transplanted into the Kir4.1-expressing donors, after which the animals were cultured in ivermectin. Visualization of donor tissue indicated a suppression of hyperinnervation, with most ectopic eyes completely failing to innervate the host (only 3 of 24 hyperinnervated; *P* <0.01) (Fig. 3b,bi). This result reveals that it is the voltage state of the host (not the donor tissue) that is crucial for the effect because introduction of the potassium channel mRNA into the host alone was sufficient. To further bolster the K^+^ and Cl^–^ data, we next used a sodium-based method. The paralytic tricaine mesylate inhibits sodium currents in affected cells, thus causing hyperpolarization, and has been shown to reduce regeneration of central nervous system (CNS) structures in *Xenopus* tadpoles [67]. Culturing tadpoles in this compound in conjunction with ivermectin following transplantation revealed that, as with Kir4.1, innervation was reduced to the levels found in untreated tadpoles (1 of 34; *P* <0.01) (Fig. 3c, ci). Taken together, these 3 results support bioelectrical signaling as a key regulator of the hyperinnervation phenotype.

Thus, depolarization through ivermectin is convenient and sufficient to drive the hyperinnervation effect, while hyperpolarization through chloride level alteration, potassium channel misexpression, or sodium channel blockade all inhibit the effect of ivermectin. Together, these results provide evidence that hyperinnervation by ectopic eyes is not strictly dependent on chloride channel activation specifically, but rather is a consequence of membrane potential depolarization, which can be induced by a variety of ion movements.

### *V*_mem_ Change Regulates Host Innervation Signals Through 5-HT Pathways

**Fig. 4:**
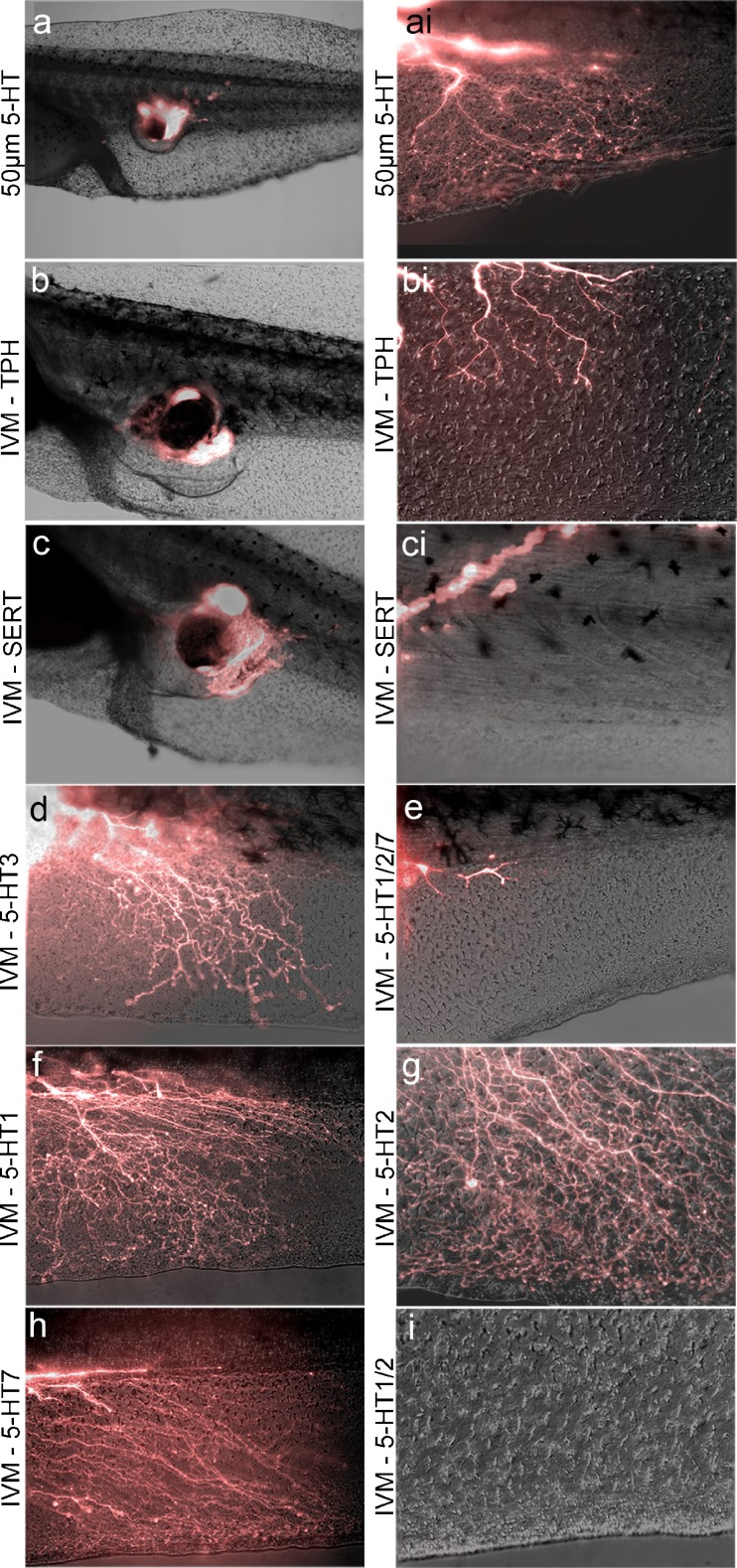
Membrane voltage control of ectopic eye innervation functions through serotonin (5-HT) signaling. (a) Supplementing the *Xenopus* media with 5-HT can induce hyperinnervation, even in the absence of membrane voltage alteration. (b) Disrupting 5-HT production in *Xenopus* embryos through *para*-chlorophenylalanine (TPH) exposure inhibits ectopic eye innervation of host animals. (c) Inhibition of the 5-HT transporter (SERT) with fluoxetine blocks hyperinnervation in response to membrane depolarization. (d) Selectively blocking 5-HT receptor 3 (5-HT3) with exposure to tropisetron has no effect on ectopic eye innervation of the host. (e) Inhibition of the 5-HT receptors 1, 2, and 7 (5-HT1, 2, 7)_with the broad-spectrum antagonist metergoline abolishes ectopic eye innervation of the host. (f) Disruption of 5-HT receptor 1A and 1B (5-HT1) with the antagonist cyanopindolol does not inhibit host innervation by ectopic eyes. (g) Inhibiting 5-HT receptor 2A (5-HT2) with the compound altanserin has no effect on ectopic eye innervation of the host. (h) Selectively blocking 5-HT receptor 7 (5-HT7) with exposure to SB258719 does not inhibit ectopic eye innervation of the host. (i) Using a combination of altanserin and cyanopindolol, innervation of hosts by ectopic eyes could be suppressed, indicating a downstream role of 5-HT receptors 1 and 2 (5-HT1/2). IVM = ivermectin

The transduction of membrane voltage changes to downstream signaling cascades can occur through a variety of mechanisms [68]. In a suppression screen of known candidate transduction mechanisms (data not shown), we identified 5-HT signaling as a possible candidate for signal transduction in ectopic eye innervation. To test the role that host 5-HT may play in the hyperinnervation process, tadpoles were cultured in media supplemented with 50 μM 5-HT. Under these conditions, 17 % of the ectopic eyes hyperinnervated the hosts, a significantly higher rate than that observed in untreated transplants (*n* =62; *P* =0.03) (Fig. 4a, ai). Thus, elevated serotonergic signaling could mimic the effect of ivermectin in a gain-of-function experiment. Further, to assess quantitatively the level to which embryos sequestered extracellular 5-HT, stage 41 embryos were analyzed for 5-HT content. WT embryos contained 309 ±93 pg per animal (*n* =4) and no significant changes in overall content were observed in response to ivermectin exposure, with an observed value of 276 ±82 pg per animal (*n* =4). Adding 5-HT to the media revealed a bioaccumulation of 5-HT in embryos, with values exceeding 344 ±146 μg per animal (*n* =4), confirming the ability of *Xenopus* cells to take up 5-HT from their surroundings.

In a loss-of-function experiment, embryos were raised from the 1-cell stage in *para*-chlorophenylalanine, which binds the enzyme tryptophan hydroxylase and inhibits 5-HT synthesis [69]. *Para*-chlorophenylalanine exposure resulted in an incomplete but significant 20 % reduction of ectopic eye innervation in the presence of ivermectin (6 of 28; *P* =0.04), further supporting a role for 5-HT in the observed phenotypes (Fig. 4b,bi). Finally, tadpoles receiving transplants were raised in a combination of ivermectin and the selective 5-HT reuptake inhibitor fluoxetine [70], which blocks the movement of 5-HT through the 5-HT transporter (SERT) and has been extensively tested in the *Xenopus* system [56, 71–75]. In the presence of fluoxetine, none of the resultant tadpoles were hyperinnervated, completely blocking the effect of ivermectin exposure (*n* =29; *P* <0.01) (Fig. 4c,ci). We conclude that the dynamics of serotonergic signaling are involved in mediating the effect of *V*_mem_ upon ectopic nerve growth, and to flesh out more fully the mechanistic pathway leading from *V*_mem_ change to hyperinnervation, we next asked how the 5-HT levels might be ascertained by cells.

The presence of extracellular 5-HT can be read by cells through a family of 7 membrane-bound 5-HT receptors [76], and we sought to determine whether specific 5-HT receptor family members were implicated in the hyperinnervation effect [77, 78]. We began by inhibiting the 5-HT_3_ receptor with the compound tropisetron, and 5-HT_1_/5-HT_2_/5-HT_7_ receptors with the compound metergoline, in eye-transplanted embryos exposed to ivermectin. Tropisetron exposure had no effect on hyperinnervation (Fig. 4d), with rates identical to those of ivermectin exposure alone (12 of 34; *P* =0.55), but metergoline had a striking antagonistic effect, with none of the treated animals demonstrating hyperinnervation (*n* =31; *P* <0.01) (Fig. 4e). The 5-HT_1_, 5-HT_2_, and 5-HT_7_ receptors were then targeted individually with cyanopridolol, altanserin, and SB 258719, respectively. However, none of these agents alone resulted in reduced levels of hyperinnervation (*n* ≥29; *P* ≥0.44 in each case) (Fig. 4f–h), leading us to test combinations of these compounds. Combined 5-HT_1_/5-HT_2_ inhibition with a combination of altanserin and cyanopridolol resulted in a reduction of hyperinnervation from 37 % to 10 % (*n* =30; *P* <0.01), suggesting 5-HT-1,2 receptors as likely downstream sensors of voltage changes within host animals (Fig. 4i).

### Gap Junction Communication is Required for Host Hyperinnervation

**Fig. 5:**
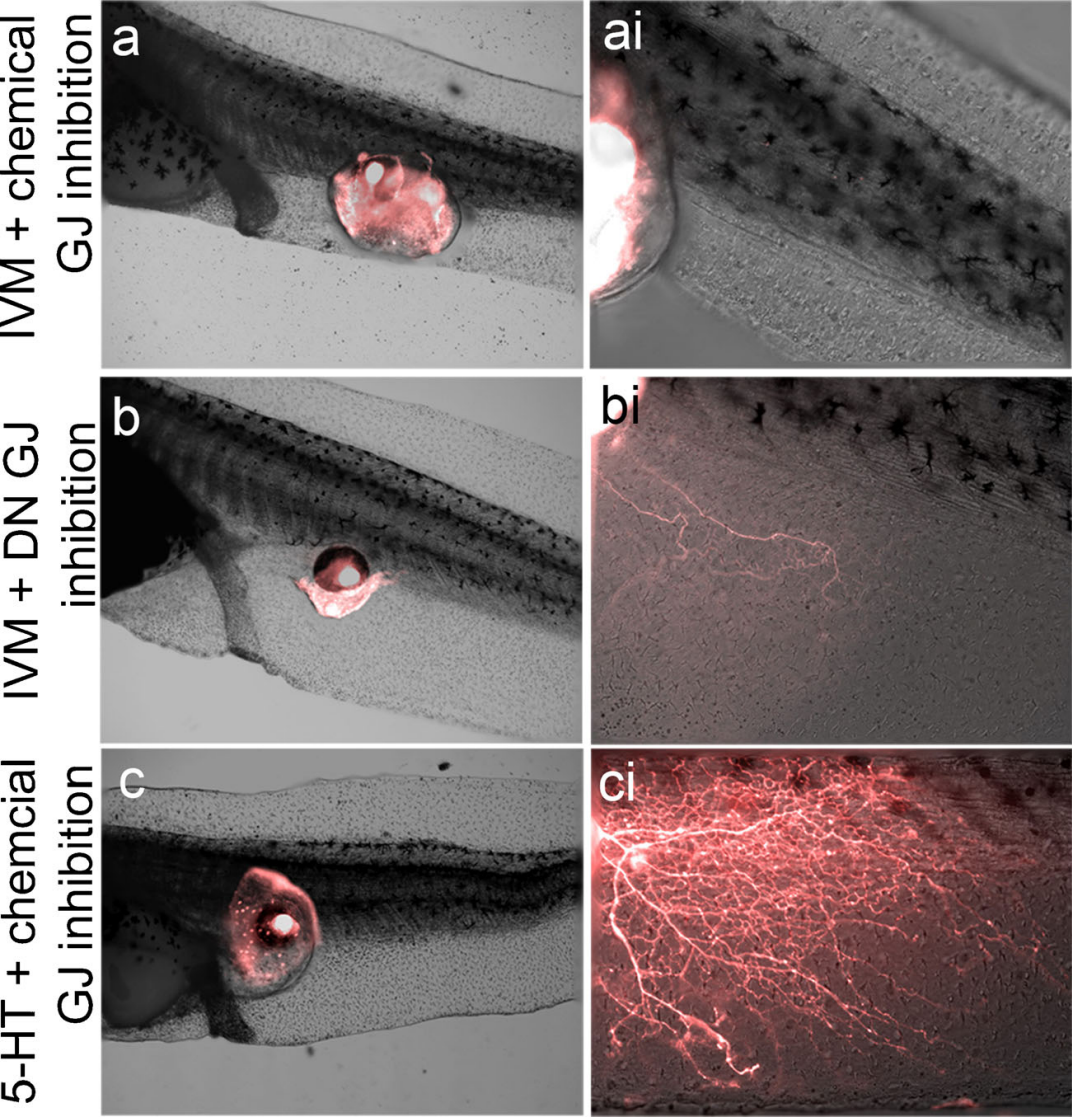
Gap junction (GJ) communication is essential for ectopic eye innervation of the host. (a) In the presence of the ivermectin (IVM), hyperinnervation by ectopic eyes could be suppressed by exposure to the GJ antagonist lindane. (b) Similar to chemical exposure, hyperinnervation by ectopic eyes in response to IVM could also be suppressed by early mRNA injections of the dominant negative (DN) GJ H7. (c) Animals treated with both the gap junction inhibitor lindane and supplemented serotonin (5-HT) resulted in hyperinnervation

5-HT can be secreted extracellularly as a signaling molecule during development, but it can also move between cells by passing through gap junctions [79, 80], along voltage gradients (towards more negatively charged cells), as occurs, for example, during left–right patterning [81–85]. Gap junctions also play a role in other examples of bioelectrically controlled morphogenetic decisions [86–91], and are expressed in a wide variety of tissue types in *Xenopus*, including epidermis, tail, lateral plate mesoderm, and neural tissue [92–94]. To determine if intercellular communication mediated by gap junctions might play a role in ivermectin-induced hyperinnervation of ectopic eyes, we inhibited gap junction channel communication with the blocker lindane [95–97]. This compound was a potent inhibitor of ectopic eye innervation in response to ivermectin (*n* =32; *P* <0.01), with <5 % of treated animals hyperinnervating the host (Fig. 5a,ai).

To validate this result with a molecular reagent, we employed the dominant negative gap junction H7 construct. Injection of this mRNA into embryos results in the formation of nonfunctional connexin chimeras, reducing or abolishing native gap junction communication [60, 98, 99]. Embryos were injected with H7 mRNA at the 1-cell stage, resulting in ubiquitous expression, and cultured in ivermectin following transplantation of eye primordium. When donor tissue was visualized, hyperinnervation was strongly inhibited and levels were indistinguishable from those of untreated controls (3 of 38; *P* =0.41) (Fig. 5b, bi). These results confirm those of chemical gap junction inhibition, and show that gap junction communication is essential for ectopic eyes to hyperinnervate hosts in response to membrane depolarization.

Finally, we sought to determine whether 5-HT signaling was acting upstream or downstream of gap junction communication. Embryos receiving grafts were cultured in a combination of the gap junction inhibitor lindane and 50 μM 5-HT. Animals raised in these conditions and exposed to ivermectin after eye transplant demonstrated hyperinnervation in 23 % of the animals examined, a rate that was statistically indistinguishable from animals treated with 5-HT alone (*n* =31; *P* =0.38 (Fig. 5c, ci). The finding that 5-HT can reverse the inhibitory effect of the lindane suggests that 5-HT signaling occurs downstream of gap junction communication.

## Discussion

The present study demonstrates the control of innervation of host tissue by grafted sensory organs in *Xenopus* tadpoles through the modulation of membrane potential. Ectopic eyes were induced in the tails of developing embryos through the transplantation of eye primordia taken from fluorescently labeled donors, allowing the visualization of donor neurites in the host. Ectopic eyes normally display low levels of innervation within the host fin; however, when host cells are depolarized, transplanted eyes show a drastically increased amount of branched innervation, which spreads throughout the fin and trunk. This effect was biphasic (animals demonstrated either no innervation or drastic hyperinnervation), with 40 % of treated animals examining the phenotype. This likely underestimates the true penetrance because the efficiency is limited by: the dilution of our reagents to low concentrations that will not interfere with normal development, physiological variability among the hosts, and tissue/location variation inherent in manual microtransplantation. Interestingly, the host’s native nerves, and tissues transplanted to their normal location in the head, did not hyperinnervate in response to depolarization. This may be owing to retinal ganglion pathfinding cues in the brain over-riding the bioelectric signaling induced by ivermectin (but being absent outside of the head, which allows nerve growth to respond to bioelectrical properties instead), or, more broadly, that tissue becomes more sensitive to bioelectric signals when it is located in an anatomically incorrect location. These findings highlight a novel role (regulation of innervation paths) for bioelectric signaling via control of transmembrane resting potential (distinct from prior work using electrodes to understand cell responses to externally applied electric fields [39, 40]) and identify a novel target for studies in molecular medicine seeking to implant and connect bioengineered sensory structures.

Hyperinnervation can be inhibited through multiple distinct methods of hyperpolarization, suggesting the effect is a more general response to membrane voltage depolarization itself and not limited to the action of a single ion channel or ion type. This finding confirms similar results in the molecular bioelectricity of cancer [56, 100], left–right asymmetry [58, 84, 101], planarian regeneration [102], and tail/limb regeneration [103, 104]. In all of these cases (including those that were host-specific, such as K^+^ channel mRNA overexpression), it was shown that a wide variety of distinct methods and ion fluxes could be used to induce the same patterning and cell behavior response provided the resting potential and its distribution were altered appropriately. Thus, hyperinnervation is likewise a true bioelectric effect in which cells sense their neighbors’ *V*_mem_.

Experiments with reagents targeting serotonergic signaling implicated a role for 5-HT in ivermectin-induced hyperinnervation. In mammalian systems, 5-HT has been increasingly recognized as a key player in both axon guidance and neurogenesis. Decreases in 5-HT levels have been shown to inhibit neurogenesis in the brain of adult rats [105], and retinogenesis in amphibians [106]. In addition, 5-HT signaling has been shown to affect the patterning of both the developing visual system and the isothalamus [107, 108]; as in our results, these studies also implicated the 5-HT_1_ family as key signaling cascade member. This is supported by several recent studies showing that serotonergic signaling modulates neural guidance directly [107, 109, 110]. Here we show that extracellular 5-HT availability can drive hyperinnervation and present data that implicate membrane potential as an upstream regulator of 5-HT availability developing nervous system. It is not yet clear if this hyperinnervation is achieved through increased axon outgrowth, retinal ganglion cell proliferation, or decreased cell death, and future studies will be necessary to differentiate between these possibilities.

The current findings also support a growing body of literature linking gap junction to CNS development; a number of studies have shown that gap junctions are key players in regulation of neural connectivity and recognition among neurons [111–114]. Here, we report gap junctional communication as a mediator of ivermectin-induced hyperinnervation. This result was consistent with a role for the intracellular transport of 5-HT between cells according to voltage gradients, where it is then stored in negatively charged cells. In many ways this model mirrors that of left–right axis specification, which requires 5-HT transport, followed by localization, in early *Xenopus* development [83]. Beyond the results presented, additional evidence also supports the role of gap junction expression and communication in the proper patterning of the developing CNS, as connexins and innexins have been reported to regulate neural proliferation in the neocortex and network morphology of cells in culture [115–119], as well as determining self *versus* not-self in neural connectivity [120]. 5-HT has also been shown to have a role in gap junction signaling. In addition to moving through gap junctions in response to voltage difference, 5-HT also appears to have a feedback mechanism by which it can affect the open and closed state probabilities of gap junctions between adjoining cells [121, 122]. The feedback between 5-HT movement among cells (via gap junctional communication), as well as the overall regulation of intracellular 5-HT levels (via SERT), establishes the possibility of forming signaling loops with rich emergent dynamics. An understanding of these complex dynamics will likely be required to gain control of nerve growth in biomedical applications.

**Fig. 6:**
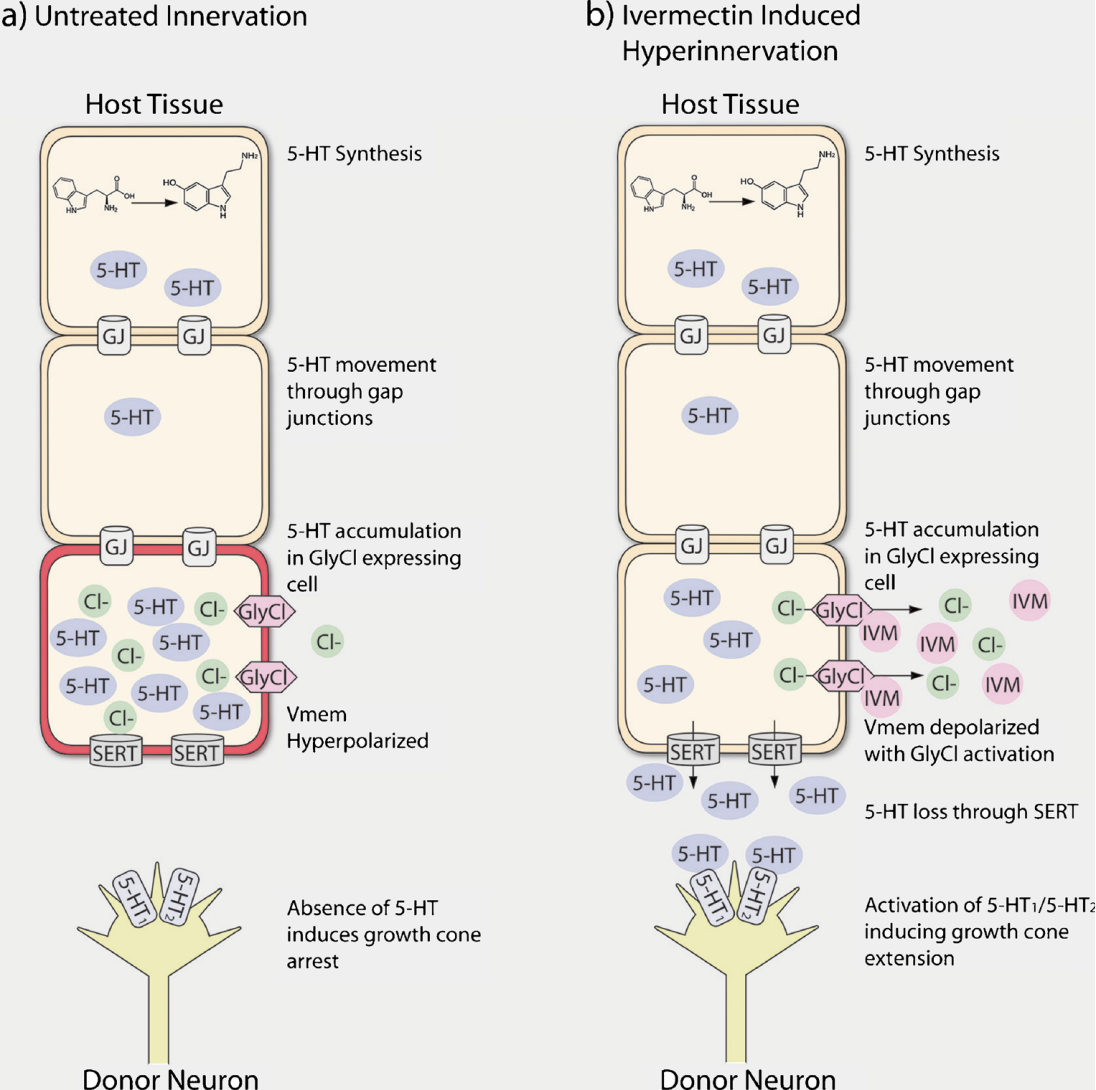
Model of ectopic eye innervation in response to membrane voltage changes. (a) In untreated animals, serotonin (5-HT) is produced across time space during development. The positively charged 5-HT moves between cells via gap junctions and accumulates in negatively hyperpolarized cells, which act as sinks for the molecule. In the absence of extracellular 5-HT, the 5-HT receptors of ectopic eye neurons are not activated and the growth cones show minimal extension. (b) In treated animals 5-HT also accumulates in negatively hyperpolarized cells. However, exposure to ivermectin (IVM) activates glycine-gated chloride channels (GlyCl), allowing chloride to exit the cell along its concentration gradient, depolarizing the cell. In response to depolarization, the accumulated 5-HT diffuses out of the cell via the 5-HT transporter (SERT). Extracellular 5-HT then binds 5-HT_1/2_ receptors on the surface of ectopic eye retinal ganglion cells, leading to extension of the growth cone

Integrating all of the data, we suggest one possible model that accounts for all of the observed results (Fig. 6). In untreated animals, 5-HT synthesis begins following depletion of maternal 5-HT stores [83], and is distributed across the embryo. As a positively charged molecule that can pass through gap junctions, 5-HT then accumulates in strongly negative (hyperpolarized) cells, which sequester the molecule from the surrounding tissue (mirroring the normal reuptake function of SERT). In the absence of extracellular 5-HT, ectopic axons receive no signaling from 5-HT receptors on their surface and as a result exhibit very limited growth cone extension (Fig. 6a). In animals treated with ivermectin, the 5-HT sequestering cells are depolarized in response to glycine-gated chloride channel activation and the subsequent loss of chloride ions [56]. In the absence of their normal hyperpolarization 5-HT translocates into extracellular space via SERT, where it binds 5-HT_1/2_ receptors on the surface of donor retinal ganglion cells, inducing growth cone elongation and hyperinnervation of host tissue (Fig. 6b). This model in many ways matches what has been reported in the developing visual system of mice, where retinal ganglion cells must uptake specific amounts of 5-HT from the extracellular environment for normal patterning to proceed [108].

The response of nerves to *V*_mem_ of cells in their environment (i.e., a nonautonomous effect arising from neighbors or distant cells rather than the neurons themselves) is particularly exciting because membrane potential could be regulated with extreme temporal precision, for example using optogenetic stimulation to set up standing patterns of resting potential in surrounding tissue [104, 123, 124]. This would enable bioengineers to refine technologies for sculpting neural connections in biomedical applications or in synthetic bioengineered constructs (innervated biobots [125]). Moreover, all host cells express an array of ion channels that could be modulated to produce a desired membrane potential. Unlike diffusible signals, changes in *V*_mem_ do not require gene transcription, translation, or chemical gradients; cells can rapidly depolarize or hyperpolarize with only a change in the open or closed state of specific ion channels, which can be induced by any convenient pharmacological agent (many of which are already approved for human medical use and are increasingly recognized as “electroceuticals” [52, 53]). Thus, our data suggest a new class of ion channel drug-based strategies for directing transplanted or regenerating neurons in biomedical applications via sculpting of bioelectrical patterns in surrounding tissues.

## Electronic supplementary material

Below is the link to the electronic supplementary material.

Supplementary Figure 1 Chloride channel activation does not alter native *Xenopus* innervation. (a) Immunohistochemistry to visualize the neuronal marker acetylated tubulin shows wild-type optic nerve morphology (white arrow), the characteristic chevron pattern of the somites, and the lateral line (dotted line) running anterior to posterior in *Xenopus* tadpoles. (b) Treatment of animals with the glutamate-gated chloride channel ivermectin (IVM) throughout development does not alter normal development of the optic nerve, somites, or lateral line. (c) Presence of an ectopic eye arising from eye primordium transplantation does not alter native innervation in the host animal. (d) While IVM exposure results in hyperinnervation arising from donor tissue following transplantation, host innervation remains unchanged. (e) Concentric circles with increasing radii of 50 μm were applied to images of wild-type and IVM-treated tails for innervation comparison by Sholl analysis. (f) Sholl analysis reveals no differences in axon number between control and IVM-treated animals (*n* =6 for each treatment, 2-way analysis of variance *P* =0.43) (JPEG 927 kb)

(PDF 1.19 mb)
