# Supplementary figures and images for: A Novel Method for Inducing Nerve Growth via Modulation of Host Resting Potential: Gap Junction-Mediated and Serotonergic Signaling Mechanisms
Source: Neurotherapeutics. 2014 Dec 2;12(1):170–84. doi: 10.1007/s13311-014-0317-7 (PMC4322068; doi:10.1007/s13311-014-0317-7)

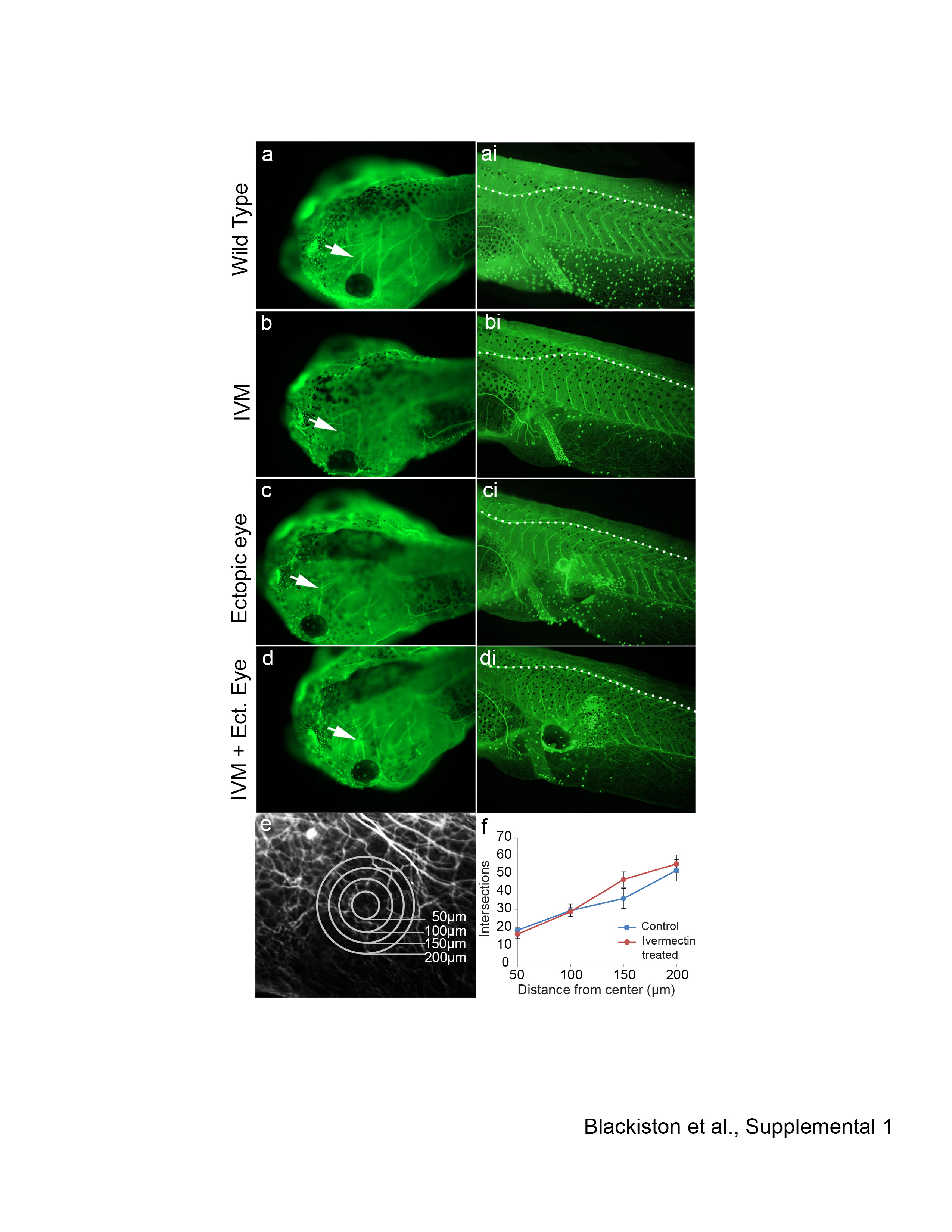

Supplement: Supplementary file 1 — Chloride channel activation does not alter native Xenopus innervation. (a) Immunohistochemistry to visualize the neuronal marker acetylated tubulin shows wild-type optic nerve morphology (white arrow), the characteristic chevron pattern of the somites, and the lateral line (dotted line) running anterior to posterior in Xenopus tadpoles. (b) Treatment of animals with the glutamate-gated chloride channel ivermectin (IVM) throughout development does not alter normal development of the optic nerve, somites, or lateral line. (c) Presence of an ectopic eye arising from eye primordium transplantation does not alter native innervation in the host animal. (d) While IVM exposure results in hyperinnervation arising from donor tissue following transplantation, host innervation remains unchanged. (e) Concentric circles with increasing radii of 50 μm were applied to images of wild-type and IVM-treated tails for innervation comparison by Sholl analysis. (f) Sholl analysis reveals no differences in axon number between control and IVM-treated animals (n =6 for each treatment, 2-way analysis of variance P =0.43) (JPEG 927 kb) [file 13311_2014_317_Fig7_ESM.jpg]
